# Group A *Streptococcus-*Induced Activation of Human Plasminogen Is Required for Keratinocyte Wound Retraction and Rapid Clot Dissolution

**DOI:** 10.3389/fcvm.2021.667554

**Published:** 2021-06-10

**Authors:** Henry M. Vu, Daniel E. Hammers, Zhong Liang, Gabrielle L. Nguyen, Mary E. Benz, Thomas E. Moran, Dustin L. Higashi, Claudia J. Park, Yetunde A. Ayinuola, Deborah L. Donahue, Ana L. Flores-Mireles, Victoria A. Ploplis, Francis J. Castellino, Shaun W. Lee

**Affiliations:** ^1^Department of Biological Sciences, University of Notre Dame, Notre Dame, IN, United States; ^2^W. M. Keck Center for Transgene Research, University of Notre Dame, Notre Dame, IN, United States; ^3^Eck Institute for Global Health, University of Notre Dame, Notre Dame, IN, United States; ^4^Department of Restorative Dentistry, Oregon Health and Science University, Portland, OR, United States; ^5^Department of Chemistry and Biochemistry, University of Notre Dame, Notre Dame, IN, United States

**Keywords:** plasminogen, plasmin, fibrinolysis, *Streptococcus pyogenes*, live imaging microscopy, wound retraction

## Abstract

Invasive outcomes of Group A *Streptococcus* (GAS) infections that involve damage to skin and other tissues are initiated when these bacteria colonize and disseminate *via* an open wound to gain access to blood and deeper tissues. Two critical GAS virulence factors, Plasminogen-Associated M-Protein (PAM) and streptokinase (SK), work in concert to bind and activate host human plasminogen (hPg) in order to create a localized proteolytic environment that alters wound-site architecture. Using a wound scratch assay with immortalized epithelial cells, real-time live imaging (RTLI) was used to examine dynamic effects of hPg activation by a PAM-containing skin-trophic GAS isolate (AP53R^+^S^−^) during the course of infection. RTLI of these wound models revealed that retraction of the epithelial wound required both GAS and hPg. Isogenic AP53R^+^S^−^ mutants lacking SK or PAM highly attenuated the time course of retraction of the keratinocyte wound. We also found that relocalization of integrin β1 from the membrane to the cytoplasm occurred during the wound retraction event. We devised a combined *in situ-*based cellular model of fibrin clot-in epithelial wound to visualize the progress of GAS pathogenesis by RTLI. Our findings showed GAS AP53R^+^S^−^ hierarchically dissolved the fibrin clot prior to the retraction of keratinocyte monolayers at the leading edge of the wound. Overall, our studies reveal that localized activation of hPg by AP53R^+^S^−^ *via* SK and PAM during infection plays a critical role in dissemination of bacteria at the wound site through both rapid dissolution of the fibrin clot and retraction of the keratinocyte wound layer.

## Introduction

Group A *Streptococcus* (*Streptococcus pyogenes* or GAS) is a Gram-positive bacterial pathogen responsible for common human disorders, including pharyngitis, cellulitis, and impetigo, in addition to invasive and severe diseases, such as rheumatic fever, toxic shock syndrome, and necrotizing fasciitis (1–3). Annually, GAS is responsible for ~1.78 million invasive infections worldwide, and these infections cause over 500,000 deaths globally (2, 4). Invasive GAS infections at the skin and tissue levels largely arise and progress due to GAS colonization and dissemination *via* an open wound (4, 5).

A critical determinant for skin-trophic strains of GAS dissemination during infection is mediated by two GAS virulence factors, human plasminogen (hPg)-binding M-protein (PAM) and streptokinase (SK2b) (6–10). PAM is an M-protein variant that displays high affinity binding to human plasminogen (hPg) (9, 11–14). The secreted protein SK2b, from GAS subsequently activates PAM-bound hPg to hPm through cleavage of the R^561^V^562^ peptide bond and removal of a N-terminal 77-residue activation peptide (6, 11, 15–18).

hPm proteolyzes a broad array of substrates, including fibrin (Fn) clots and proteins that maintain cellular integrity (19–24). This activity allows hPm to play a role in the maintenance of cellular morphology and architecture, as well as affect cellular attachment to substrates and other cell types in local tissue. As a result, hPm has an important role in wound healing and the re-epithelialization process. When an epithelial wound occurs, a blood clot containing fibrin is formed (25). In order for keratinocytes to migrate into the wound and continue the healing process, these cells dissolve and remodel the fibrin clot by activating hPg to hPm. This process and subsequent keratinocyte migration are dependent on the urokinase-type plasminogen activator receptor (uPAR) and integrins on the keratinocyte cell surface (26, 27).

The importance of integrins in wound healing is well-known, as they are cell receptors that mediate cell attachment to the extracellular matrix (ECM), and bind with other receptors, such as uPAR (26, 28–30). Once uPAR and integrins are bound, uPAR can affect integrin conformation and affinity as well as promote intracellular signaling involved with tissue remodeling and cellular migration (30, 31). In the wound healing process, integrins, such as β1, mediate keratinocyte migration in re-epithelization of the wound (32). During infection, GAS has been shown to interact with hPg to promote bacterial invasion and internalization into keratinocytes *via* α1β1- and α5β1-integrins, where the bacteria can remain viable and avoid immune system detection (33). The inability to remove bacteria, such as GAS, from a wound can lead to a prolonged inflammatory period, which can promote an increase in matrix metalloproteases (MMPs) (34). The MMPs then degrade the ECM and prevent further wound healing. Given the interactions demonstrated with GAS/hPg and integrin β1, and the known role of integrin β1 in wound healing, integrin β1 thus serves as an important host component targeted by bacteria during invasion and dissemination.

Although cell-based studies have provided important details regarding the manner in which PAM and SK contribute to invasive GAS disease, real-time dynamics of how these virulence factors manipulate cell and tissue architecture during infection have not been critically examined. In the current study, we utilized real time live imaging microscopy (RTLI) to establish and document a GAS infection model of a keratinocyte wound and the role of the fibrin clot therein. In particular, we sought to determine the specific role of hPg activation by GAS in the context of wound remodeling and clot dissolution, using a standardized scratch-based keratinocyte wound infection model to dynamically visualize the progress of GAS infection under epithelial wound conditions. Furthermore, we established conditions to image the rate of dissolution of Fn clots during GAS infection using RTLI. Coupling the rate of fibrinolysis during GAS infection, together with the rate of keratinocyte wound remodeling, allowed the development of a unique fibrin clot-in-wound model for *in situ*-based cellular modeling during GAS infection in real-time.

## Materials and Methods

### Bacterial Cultures

The *Streptococcus pyogenes* isolate, AP53/covR^+^covS^−^ (AP53R^+^S^−^), was provided by Dr. Gunnar Lindahl (Lund, Sweden). This strain is a clinical isolate from a patient with necrotizing fasciitis with a mutation in the sensor (S) component of the two-component control of virulence (cov) responder (R)/extracellular sensor (S) gene regulatory system, *covRS* or *csrRS*, which significantly alters the expression of other virulence genes and enhances the invasive capability of the strain (35, 36). The two isogenic mutants (AP53R^+^S^−^/ΔPAM and AP53R^+^S^−^/ΔSK) used in this study have been described elsewhere (37). The GAS AP53R^+^S^−^ strains were grown in Todd-Hewitt broth at 37°C for 16–18 h prior to RTLI and cytotoxicity studies.

### Epithelial Cell Culture

HaCaT human epithelial keratinocytes (38) were provided by Dr. Victor Nizet (UCSD). The cells were added to Dulbecco's Modified Eagle's Medium (DMEM) (Life Technologies 11995-073)/10% heat inactivated fetal bovine serum (FBS) and incubated at 37°C with 5% CO_2_. Before experimentation, the cells were placed in 12-well tissue culture-treated plates for GAS infection to determine cell viability (Corning) and in optical dishes for RTLI (MatTek).

### Keratinocyte Infection

HaCaT human epithelial keratinocytes were grown on plates and dishes to 90% confluency, and the cells were then washed 2x with sterile PBS (pH 7.4) followed by the addition of serum-free DMEM, prior to infection. GAS cultures were incubated with 7 μg/mL of plasma hPg in sodium phosphate buffer for 1 h prior to addition to cells for infection conditions that included hPg and GAS. A multiplicity of infection (MOI) 5 of bacteria per host cell was used for the studies with AP53R^+^S^−^ and its isogenic mutant GAS strains for fixed microscopy and host response measurements. The infected cells were incubated at 37°C with 5% CO_2_ over various times (ranging from 2 to 10 h) for RTLI microscopy.

### Ethidium Homodimer Cell Death Assay

HaCaT cells were infected as described above. After infection, the supernatants from the 12-well plates were collected from individual wells, and each well was washed with sterile 2x PBS. The cells were then incubated with 4 μM ethidium homodimer (Molecular Probes) in PBS at room temperature in the dark. Fluorescence measurements were obtained using a plate reader set to 528 nm excitation and 617 nm emission with a cessation value of 590 nm. To account for variability in the total number of cells per well, 0.1% (w/v) Saponin (Sigma) was added to each well-followed by incubation with gentle agitation for 20 min at room temperature, while protected from light. A second fluorescent reading for each plate was taken using the same settings. The percent membrane permeabilization was calculated individually for each well by dividing the initial plate reading (post-ethidium homodimer addition) by the second plate reading (post-Saponin addition). Each experimental condition was performed in triplicate. ANOVA and Tukey's/Holm–Šídák multiple comparisons tests were used to determine significance among experimental conditions with an α-value of 0.05. The average and standard deviation values under each condition were graphed in Graphpad Prism.

### Real-Time Live Imaging

For the wound/clot infection model, an inverted Nikon Eclipse Ti-E microscope fitted with an environmental chamber (37°C with 5% CO_2_) with 20x and 60x objectives, and a Perfect focus system (PFS) was employed. An iXon Ultra 897 electron multiplying charge-coupled device (Andor) or a Neo sCMOS (Andor) were used to capture the images. The images were analyzed and reconstructed by ImageJ/FIJI (NIH). Experiments were performed at least in triplicate for each treatment.

### RTLI of a Wound Infection Model With GAS and hPg

HaCaT cells were seeded onto optical dishes and prepared similarly to the above GAS infection conditions. A scratch assay using a 27 g syringe needle was performed to establish a model of a keratinocyte wound. Bacteria were then added at MOI 5, followed by 7 μg/mL of hPg, or 7 μg/mL of exogenous hPm (ERL) as a control. This MOI 5 was used to ensure that the imaging field would not be overwhelmed with bacteria during imaging. Images were taken every 10 min for 10 h using the 20x objective. To measure wound retractions in the experiments, measurements were made at edges of the wound at the top, middle, and bottom for each wound treatment *via* ImageJ at t = 0 or at the end of the imaging run (t = 10 h for different GAS strains and/or hPg; t = 2 h for exogenous hPm). Experiments were performed at least in triplicate for each treatment to confirm overall phenotype, and one representative live image was quantified for comparative display. Statistical analysis was done by using Graphpad Prism 9.0. To determine significance, an ANOVA and a *post-hoc* The Tukey's/Holm-Šídák multiple comparisons test were used to compare the means of each wound treatment. *P*-values of <0.05 were considered to be significant.

### SDS-PAGE and Western Blotting

Wounds in HaCaT cultures and subsequent infections with GAS were carried out as described earlier. Following infection, HaCaT cells were lysed for 20 min on ice. Total protein concentrations for each lysate sample were determined using a bicinchoninic acid (BCA) assay (Pierce) with bovine serum albumin (BSA) as the protein standard. The data were normalized prior to loading on a 4–15% gradient tris-glycine polyacrylamide gel (Bio-Rad). The proteins were transferred to polyvinylidene difluoride (PVDF) membranes primed in methanol. The resulting membranes were blocked for 1 h at room temperature in 10% skim milk powder in Tris-buffered saline/0.1% Tween 20 (Sigma, TBST), pH 7.4, followed by overnight (4°C) incubation with polyclonal anti-integrin β1 (Thermo-Fisher) or monoclonal anti-β-actin (Sigma; Clone AC-15) as 1° antibodies. The membranes were then soaked for 45 min in TBST and incubated with a corresponding horseradish peroxidase (HRP)-conjugated 2° antibody (Santa Cruz Biotechnology) for 2 h. The membranes were again soaked for 45 min in TBST, and incubated with ECL chemiluminescence reagent (Pierce) prior to imaging using an Azure Biosystems c600 imager.

### Immunofluorescence Staining and Imaging

The cells were plated on sterile glass coverslips in 6-well plates and wounds and GAS infections were performed as described above. After infection, the cells were washed in cold PBS and fixed for 2 h at room temperature in 4% (wt/vol) paraformaldehyde/PBS. The coverslips were washed in cold PBS and blocked overnight at 4°C in PBS with 1% (wt/vol) normal goat serum (Invitrogen)/2% (vol/vol) Triton X-100 (Dow/Sigma-Aldrich)/0.5% (vol/vol) Tween 20. The cells were washed with PBS for 20 min and incubated with anti-integrin β1 in blocking solution at 4°C overnight. After washing, the 2° antibody, goat-anti-rabbit IgG AlexaFluor 488 (Invitrogen), was added and allowed to incubate for 2 h at room temperature. The coverslips were then washed in PBS for 1 h, followed by incubation with 4′,6-diamidino-2-phenylindole (DAPI; Cell Signaling Technology) nuclear stain and rhodamine-phalloidin (Thermo-Fisher) actin stain in blocking buffer at room temperature. The coverslips were again washed with PBS, and were then mounted on glass slides using Fluoromount-G (Southern Biotech), and allowed to set prior to sealing and imaging on the Nikon Eclipse Ti-E microscope. During imaging, Z-stacks were taken every 0.3 μm for 30 μm. The relative fluorescence values (mean gray values) of the antibody for integrin β1 were measured using ImageJ/Fiji. The boundaries of the wound for multi-component imaging were identified manually, as the DIC images of the keratinocyte wound layer allow for clear images of the boundary front. The boundary was delineated with dashed lines only in our static image to show the location of the initial wound when the fibrin clot portion was added. The boundaries served as a consistent tool to measure the fluorescence of a wound from the basal end to the apical side of HaCaT cells across a Z-stack. The mean gray values of an individual Z-stack were then aggregated to form a fluorescence value for that specific image. The aggregated fluorescence values for each Z-stack were then graphed from the basal to apical end using Graphpad Prism.

### RTLI of a Fn Clot Infection Model With GAS and hPg

Human Fibrinogen free from hPg and von Willebrand Factor *via* affinity chromatography and analyzed on SDS-PAGE gel as a homogenous sample of human fibrinogen that is 100% clottable (Fg; 1 g/mL; Enzyme Research Laboratory) in PBS, pH 7.4, was added to an empty optical dish. Human thrombin (5 NIH units/mg; Sigma Aldrich) was added to convert Fg to fibrin (Fn). The dish was placed in an incubator at 37°C with 5% CO_2_ for 1 h. DyLight 488 NHS-Ester, a fluorescent labeling reagent (50 μg/mL in PBS), was added to the Fn clot and allowed to incubate for 2 h in the dark at room temperature. After 2x PBS washes to remove any unbound DyLight 488 NHS-Ester, DMEM media without FBS was added to the Fn clot. Bacteria and hPg were prepared as described in the RTLI infection model and added to the clot. Images were taken every 10 min for 10 h at 60x objective in the FITC channel with a 480/30 nm excitation filter and 535/45 nm filter. Experiments were performed at least in triplicate for each treatment.

### *In situ*-Based Combination Fibrin Clot-in-Wound Model for RTLI

HaCaT cells were seeded onto optical dishes and prepared similarly to the GAS infection conditions above. To mimic the wound, a scratch was made with a 27 g syringe needle through the middle of the optical dish. Fg (1 mg/mL) in PBS, pH 7.4 was converted to Fn by thrombin (5 NIH units/mg). The optical dish was then placed in an incubator at 37°C with 5% CO_2_. DyLight 488 NHS-Ester (50 μg/mL in PBS), was added to the Fn clot and incubated for 2 h in the dark at room temperature. After 2x PBS washes to remove any unbound Dylight 488 NHS-Ester, the Fn clot was moved *via* pipette tip to the scratch wound site to create a wound/clot model. Only the Fn clot is labeled with Dylight 488 NHS-Ester. Bacteria and hPg were prepared as described in the RTLI model and added to the clot. Images were taken every 10 min for 10 h at 60x objective in the FITC channel with a 480/30 nm excitation filter and 535/45 nm filter. Experiments were performed at least in triplicate for each treatment.

## Results

### hPm and GAS Activation of hPg Cause Rapid Wound Retraction of Keratinocytes

**Figure 1:**
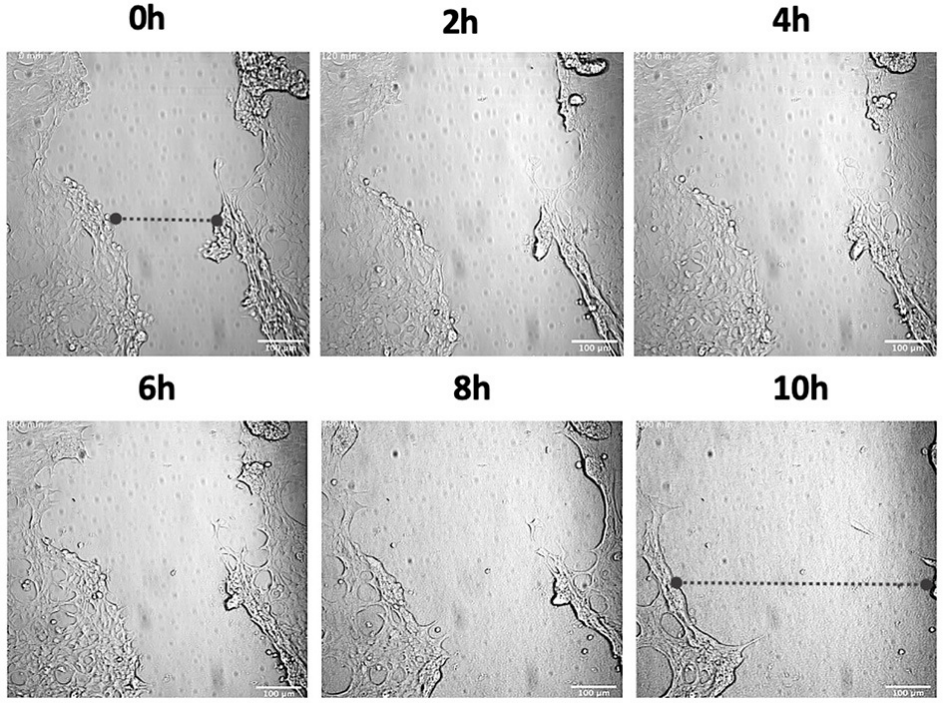
Activation of hPg by AP53R^+^S^−^ causes wound retraction of keratinocytes. Scratch wounds were produced on confluent HaCaT keratinocytes using a 27 g needle prior to incubation with GAS. The cells were then incubated with AP53R^+^S^−^ at MOI 5 along with the addition of 7 μg/ml hPg prior to live imaging. Images were obtained every 10 min for 10 h for the duration of the imaging experiment. Time-lapsed images of the experiment are shown here (see Supplementary Video 1). The dashed lines show the approximate width of wound at the start and end of the experiment.

**Figure 2:**
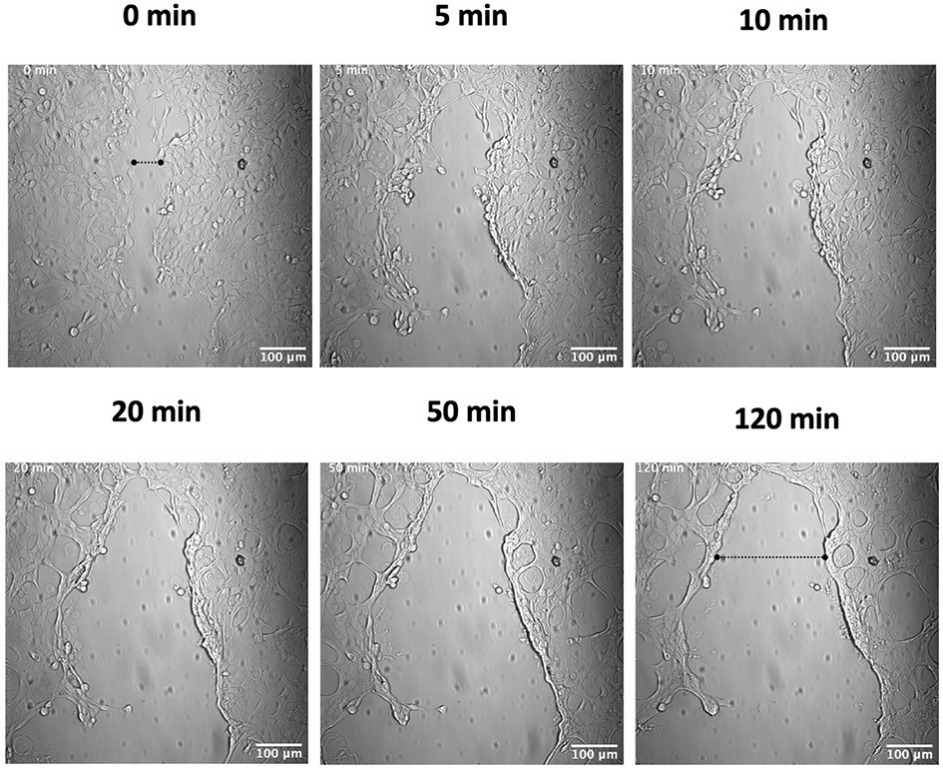
Plasmin causes a rapid wound retraction of keratinocytes. Scratch wounds were produced as in Figure 1, and cells were then incubated with 7 μg/ml of hPm and analyzed by live imaging. Images were obtained every 2.5 min for 2 h for the duration of the imaging experiment. Time-lapsed images of the experiment are shown here (see Supplementary Video 2). Dashed lines show the approximate width of the wound at the start and end of the experiment.

**Figure 3:**
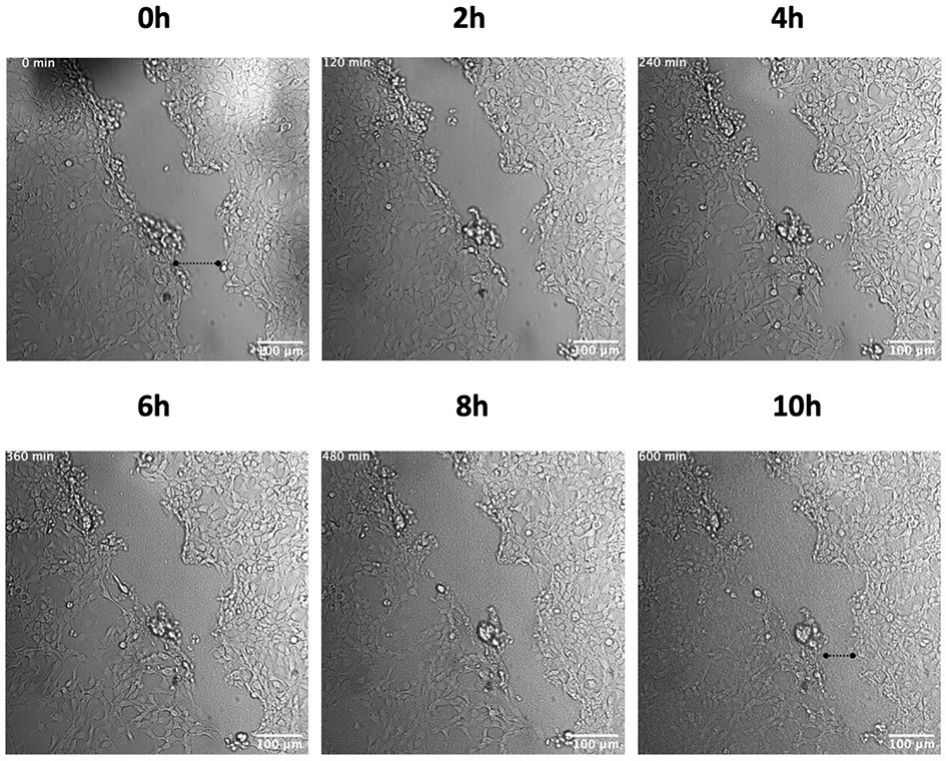
AP53R^+^S^−^ does not induce retraction of a keratinocyte wound in the absence of hPg. Scratch wounds were produced as in Figure 1. The cells were then incubated with AP53R^+^S^−^ at MOI 5 prior to live imaging. Images were obtained every 10 min for 10 h for the duration of the imaging experiment. Time-lapsed images of the experiment are shown here (see Supplementary Video 3). Dashed lines show the approximate width of wound at the start and end of the experiment.

**Figure 4:**
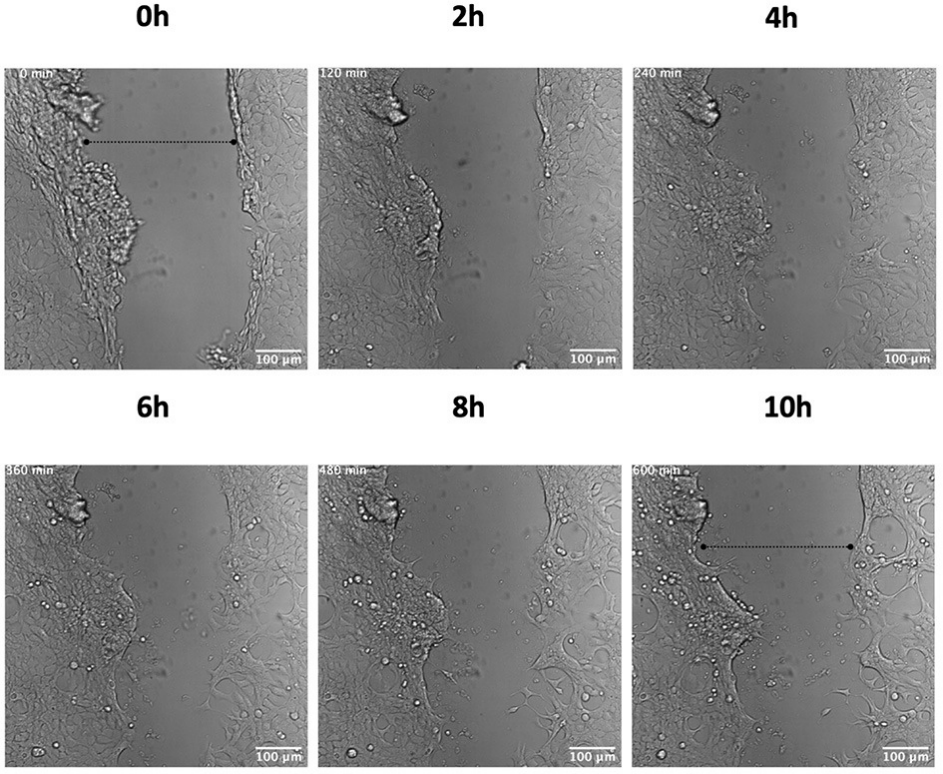
hPg does not cause keratinocyte wound retraction in the absence of GAS. Scratch wounds were produced as in Figure 1. The cells were then incubated with 7 μg/ml hPg prior to live imaging. Images were obtained every 10 min for 10 h for the duration of the imaging experiment. Time-lapsed images of the experiment are shown here (see Supplementary Video 4). Dashed lines show the approximate width of wound at the start and end of the experiment.

**Figure 5:**
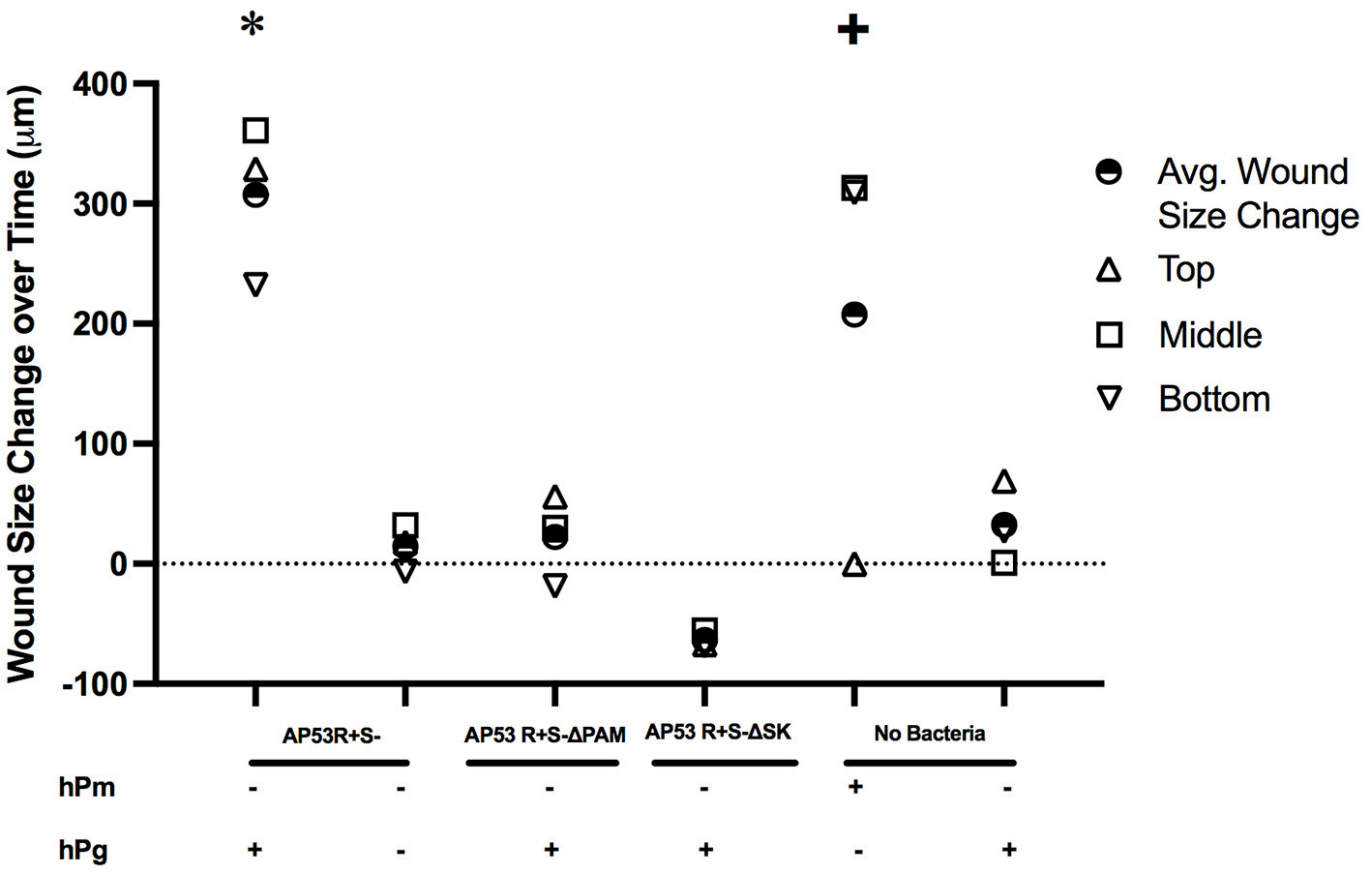
Measurement of keratinocyte wound retraction observed in live imaging by activation of hPg by AP53R^+^S^−^ and hPm. Wound assay measurements for all treatments were measured as the change in the size of the wound at the initial start of the imaging (time = 0 h) to the endpoint (t = 10 h for GAS and hPg treatments; t = 2 h for hPm). Measurements were made at edges of the wound at the top, middle, and bottom for each wound treatment *via* ImageJ. Mean wound size changes for each treatment for the three uniform wound locations are calculated and also shown as indicated. The overall *p*-values were determined by ANOVA (*p* < 0.001). The Tukey's/Holm-Šídák multiple comparisons test were performed to compare each experimental treatment. * indicates statistical significance (*p* < 0.05) of AP53R^+^S^−^ and hPg compared to all of the other treatments. + indicates statistical significance (*p* < 0.05) of hPm compared to AP53R^+^S^−^ ΔSK and hPg.

In order to investigate the role of hPg activation by GAS in cellular wound remodeling, we established a keratinocyte-based scratch assay wound system to observe the effects of GAS infection on epithelial wound interfaces. We utilized HaCaT cells seeded to 90% confluency and a 27 g needle to generate a wound furrow prior to addition of GAS. The cells were then infected with either AP53R^+^/S^−^ or isogenic variants at MOI 5. We first imaged AP53R^+^S^−^ infections at the wound furrow for 10 h. In the presence of GAS and 7 μg/mL of hPg, wound retraction commenced at ~8 h p.i. (Figure 1; Supplementary Video 1). We observed a similar rapid retraction event when exogenous hPm alone (without GAS) was added to a wound (Figure 2). However, direct addition of hPm resulted in similar wound retraction events as early as 5 min post-hPm addition (Figure 2; Supplementary Video 2). The time difference for the retraction to begin in with GAS and hPg (beginning ~8 h), compared to the exogenous hPm experiment (~5 min), was likely due to the conversion time needed by GAS to activate hPg to hPm. This activation process by GAS requires the recruitment of hPg to the bacterial surface *via* PAM. Once bound to PAM, hPg is activated to hPm by AP53-secreted SK2b. When added individually, GAS and hPg (7 μg/mL), alone, did not cause a rapid wound retraction over 10 h (Figures 3, 4; Supplementary Videos 3,4). Representative images from each assay condition were used to quantify the overall change in wound size (Figure 5). Overall, these results demonstrate that GAS AP53R^+^S^−^ cause wound retraction of keratinocytes during infection only in the presence of hPg compared to the other treatments (*p* < 0.05). Importantly, these RTLI results reveal a significant temporal latency in the activation of hPg by GAS, as opposed to adding hPm directly to the wound.

### PAM and SK Are Required for Gas to Cause Wound Retraction in the Presence of hPg

The binding and activation of hPg by the GAS virulence factors, PAM and SK, respectively, has been implicated in promoting GAS dissemination. Once hPm is bound to the GAS surface, proteolysis of the ECM and alteration of the cellular tight junctions (TJ) can occur (39–41). To further investigate the roles of PAM and SK in creating a proteolytic environment to promote host invasion, we performed these same experiments using the isogenic GAS strains, AP53R^+^S^−^/ΔPAM and AP53R^+^S^−^/ΔSK, neither of which is capable of generating hPm. Over the course of 10 h, each of the isogenic GAS mutant strains (MOI 5) were unable to rapidly retract the keratinocyte wound in the presence of hPg (Supplementary Figures 1, 2; Supplementary Videos 8,9). The results indicate that the hPm-mediated wound retraction that was observed during AP53R^+^S^−^ infection of keratinocytes is dependent on the GAS virulence factors, PAM and SK, to promote hPm formation from hPg.

### Integrin β1 Levels Are Decreased Over Time During GAS Infection of HaCaTs in the Presence of hPg

**Figure 6:**
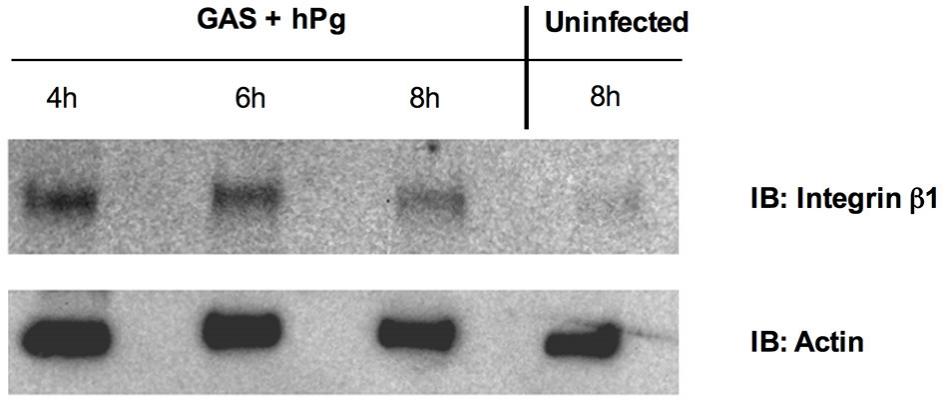
Activation of hPg by AP53R^+^S^−^ causes cytoplasmic localization and subsequent degradation of integrin β1 in a keratinocyte wound. Scratch wounds were produced as in Figure 1. HaCaT wounds were infected with AP53R^+^S^−^ at MOI 5 with 7 μg/mL of hPg. Over the course of 8 h, lysates representing cytoplasmic fractions at different timepoints were collected and assessed for the presence of integrin β1 *via* Western blotting with an antibody against Integrin β1. An uninfected wound was also assessed for integrin β1 levels in the cytoplasm, indicating Integrin β1 levels lower in uninfected cytoplasmic fractions.

**Figure 7:**
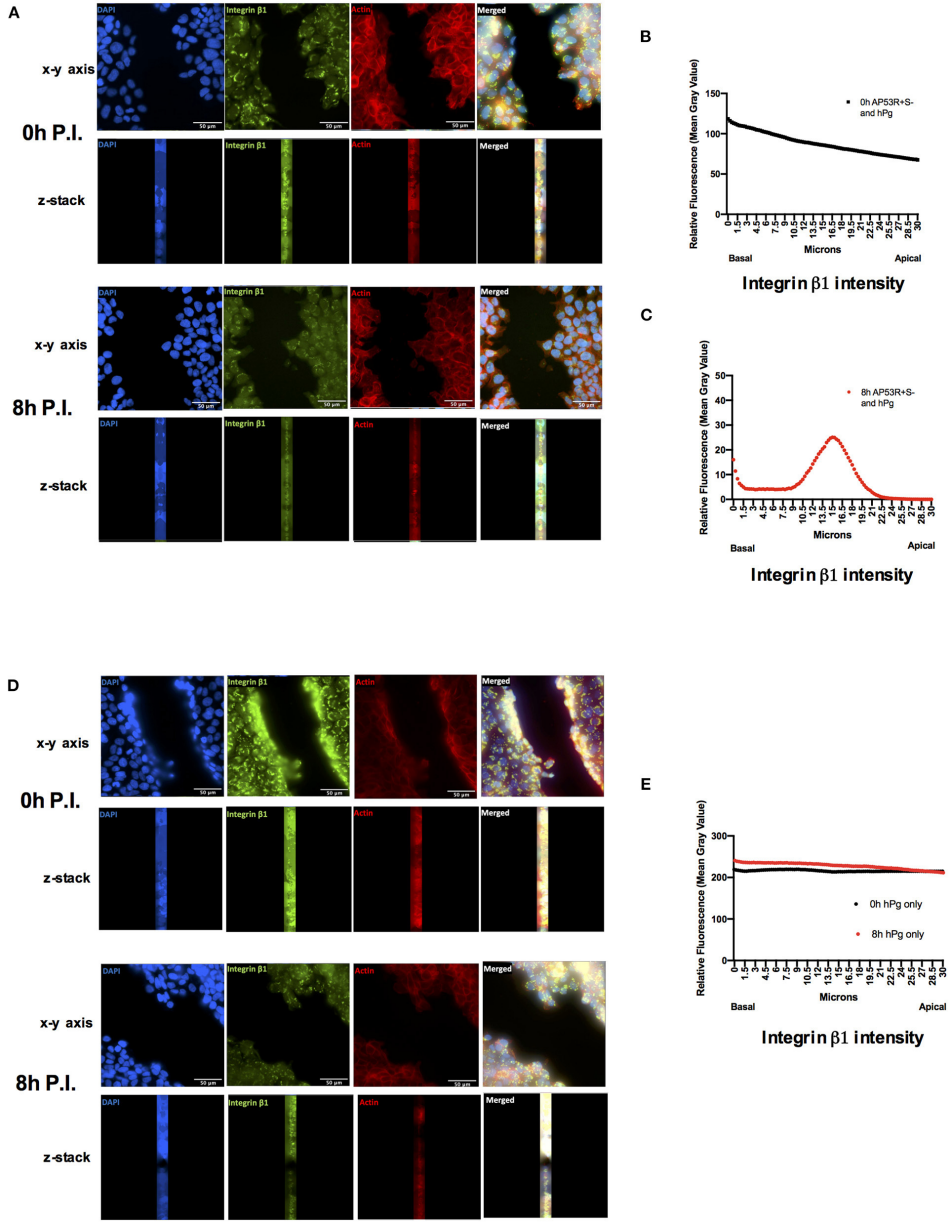
Activation of hPg by AP53R^+^S^−^ promotes relocalization of integrin β1 into the cytoplasm of a keratinocyte wound. Scratch wounds are imaged as in Figure 1. **(A)** Cells were incubated with AP53R^+^S^−^ at MOI 5 along with the addition of 7 μg/ml hPg prior to fluorescence imaging at 0 and 8 h. **(B,C)** Fluorescence readings for an antibody to integrin β1 were analyzed for each image *via* ImageJ/Fiji after infection with GAS and hPg. The black lines indicate aggregate fluorescence readings at initial infection timepoint. Red lines indicate aggregate fluorescence readings at 8 h after infection. **(D)** Cells were incubated with 7 μg/ml hPg prior to fluorescence imaging 0 and 8 h. **(E)** Fluorescence readings representative of integrin β1 were analyzed for each image *via* ImageJ/Fiji after only hPg was added. Black circles indicate aggregate fluorescence readings at initial hPg addition. Red circles indicate aggregate fluorescence readings at 8 h after hPg addition. Images were obtained every 0.3 μm for 30 μm beginning at the basal level.

To ascertain a potential target and role for GAS and hPm-mediated rapid wound retraction, we performed Western blots and immunofluorescence experiments to analyze the effects of GAS-induced hPg activation on the host protein integrin β1. Western blot analysis showed a significant loss of total integrin β1 levels between the uninfected and infected keratinocytes (Figure 6). Between 4 and 8 h post-infection, the intensity of the band corresponding to integrin β1 was shown to significantly decrease relative to the β-actin loading control. Immunofluorescence imaging of the infected keratinocyte wound revealed that GAS infection caused a relocalization of integrin β1 from the basement of the keratinocyte into the center of the cells (Figure 7). Using Z-stack imaging of the wound infection (0.3 μm for 30 μm), we observed that fluorescence readings corresponding to integrin β1 at the beginning of the GAS infection with hPg were highest at the basement of the cells (Figure 7B). Fluorescence readings at 8 h post-infection showed generally lower levels of integrin β1 with higher levels of integrin β1 corresponding to the center area of the keratinocyte, as shown by Z-stack location (Figure 7C). Uninfected cells with only hPg had integrin β1 levels consistent with uninfected control wells for up to 8 h (Figures 7D,E). When hPm was added to the wound model, integrin β1 levels trended lower with a small peak in the corresponding to the center area of the keratinocyte, similar to conditions observed with GAS and hPg, as shown by Z-stack location (Supplementary Figure 3B). When the wound was infected with AP53R^+^S^−^/ΔSK and hPg, levels for integrin β1 were consistent across the cell (Supplementary Figure 4B). Together, these results suggest that the total levels and localization of integrin β1 is significantly altered during hPm-mediated wound retraction by GAS. In particular, SK-mediated activation of hPg to hPm is required for alterations in integrin β1 during infection.

### Cell Viability of GAS Infection and hPg in Keratinocytes

We next evaluated whether the wound retraction event observed in the GAS infections was due to general host cell cytotoxicity during infection. Keratinocyte wounds with AP53R^+^S^−^ (MOI 10) and hPg (100 μg/mL) were developed and assessed for membrane permeabilization as an indication of host cell cytotoxicity. No significant differences in cell cytotoxicity were observed when HaCaTs were infected with either AP53R^+^S^−^ or the isogenic mutant strain, AP53R^+^S^−^/ΔSK, over 8 h (Supplementary Figure 5). We therefore conclude that the observed keratinocyte wound retraction observed during GAS infection is not a general result of host cytotoxicity mediated by general bacterial growth or infection, but rather is a specific consequence of hPm targeting of integrin β1 to cause the observed retraction.

### GAS Activation of hPg Results in Rapid Fn Clot Dissolution

In order to dynamically investigate the role of hPg activation by GAS-secreted SK2b on fibrin clot dissolution, we established a live-imaging fluorescent-fibrin clot model amenable to GAS infection. Fibrinogen (Fg, 1 g/mL) was converted to fibrin (Fn) in an imaging dish using thrombin (5 NIH units/mg) and subsequently incubated with DyLight 488 NHS-Ester, a fluorescent labeling reagent (50 μg/mL in PBS) to image the dissolution dynamics of a Fn clot during infection.

**Figure 8:**
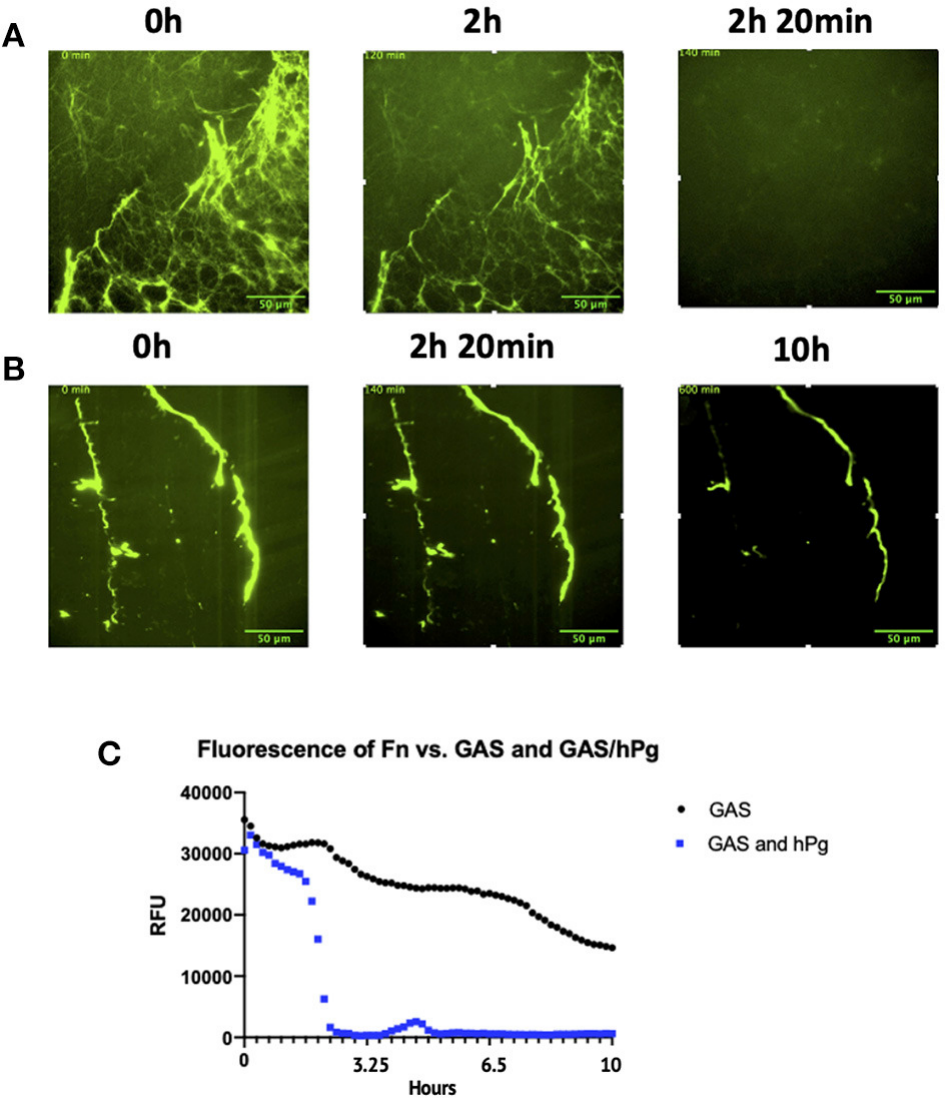
Activation of hPg by AP53R^+^S^−^ causes Fn clot lysis. Prior to live imaging, the Fn clot was incubated with: **(A)** AP53R^+^S^−^ at an MOI of 5 along with 7 μg/mL of hPg or with **(B)** only AP53R^+^S^−^ at an MOI of 5. **(C)** Fluorescence measurements reveal the dissolution or maintenance of the Fn over a 10 h period. The images were obtained every 10 min for 10 h for the duration of the imaging experiment. Time-lapsed images of experiment are shown here (see Supplementary Videos 5, 6).

At 2 h post-infection with GAS and hPg, the intensity of the fluorescence began to rapidly decrease, indicating that the clot was dissolving (Figure 8A; Supplementary Video 5). The majority of the fluorescence intensity was then lost within the next 20 min, representing complete dissolution of the clot. When only GAS was present (without hPg), the fluorescent clot remained intact, even at 10 h p.i. (Figure 8B; Supplementary Video 6). Using ImageJ/Fiji, the fluorescence intensity of the live images as a measure of Fn clot dissolution was obtained. A clear decrease in fluorescence in the presence of GAS and hPg, but not GAS alone, was observed (Figure 8C). RTLI of Fn clots infected with AP53R^+^S^−^ GAS in the absence of exogenous hPg showed a slow decrease in fluorescence over a period of 10 h with some Fn threads still intact. Finally, fibrinolysis was not detected using isogenic GAS mutants lacking SK, demonstrating that SK is essential for the rapid Fn clot dissolution observed (Supplementary Figure 6; Supplementary Video 10).

### Establishment of an *in-situ* Clot-Wound Model System for Imaging GAS Infection Dynamics

**Figure 9:**
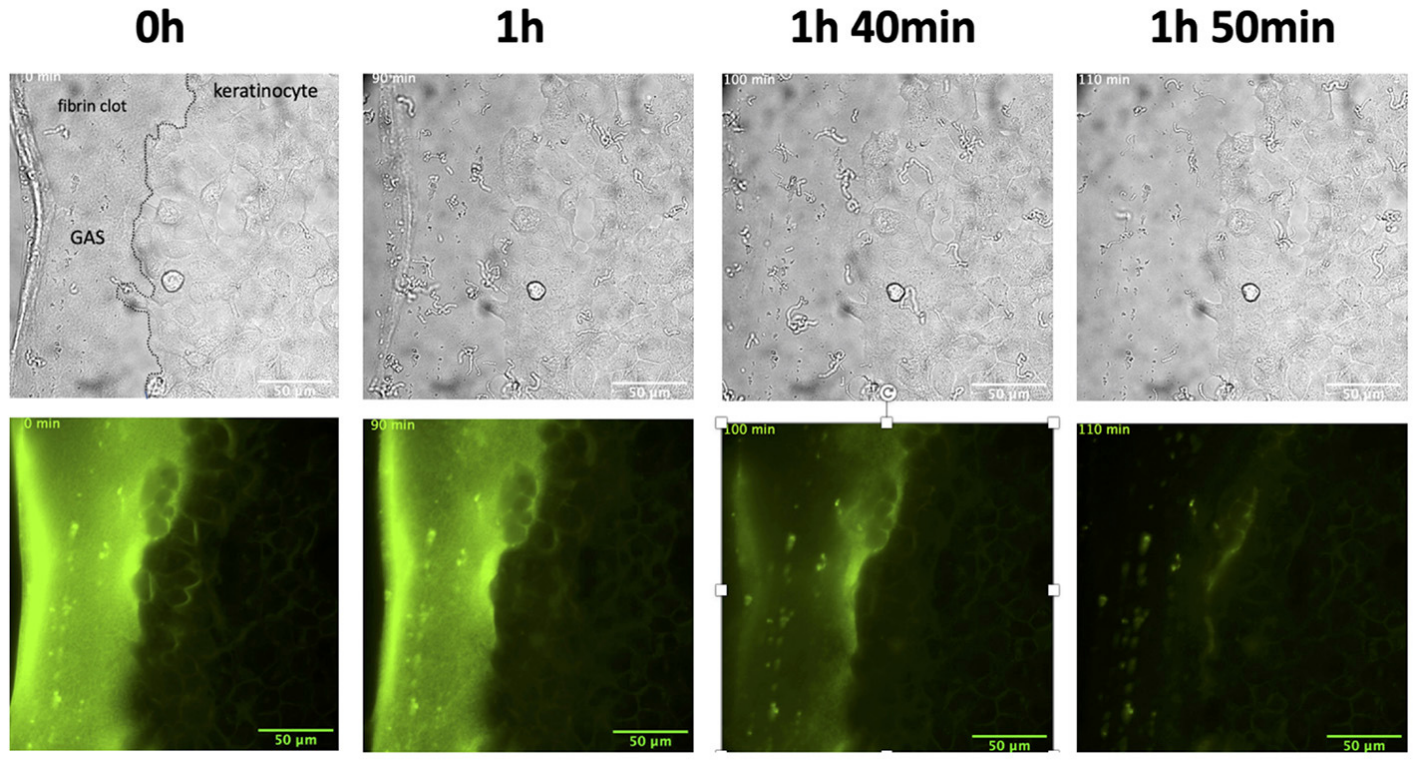
The combination wound and clot model infected with AP53R^+^S^−^ and hPg shows rapid fibrin clot dissolution in the presence of a keratinocyte wound. Prior to incubation with GAS, scratch wounds were produced as in Figure 1 and Fn was placed directly adjacent to the wound. The cells were then incubated with AP53R^+^S^−^ at an MOI 5 along with the addition of 7 μg/ml hPg prior to live imaging. Images were obtained every 10 min for 10 h for the duration of the imaging experiment. Time-lapsed images of the experiment are shown here (see Supplementary Video 7).

Lastly, we combined our keratinocyte wound and Fn models to create a unique Fn clot-in-wound model for *in situ*-based studies to observe GAS infections over 10 h. The system described represents the first combined multi-component *in situ* cellular model that approximates the local skin-tissue environment during GAS infection, and allows real-time dynamic measurements of how GAS interacts with these components during early infection. After establishment of a keratinocyte wound, a Fn clot was created inside the cleave furrow of the wound. GAS (MOI 5) and hPg (7 μg/mL) were added to initiate the infection. In this multi-component model, at 1.5 h p.i., rapid clot dissolution occurred for 20 min (Figure 9; Supplementary Video 7). The clot was completely dissolved at 2 h p.i., while the keratinocyte wound remained intact over the course of 10 h. Clearly, RTLI demonstrates that GAS hierarchically dissolves the Fn clot prior to the retraction of keratinocyte monolayers at the leading edge of the wound. Overall, our studies reveal that localized activation of hPg by GAS by SK2b bound to PAM during infection plays a critical role in bacterial dissemination at the wound site through both rapid dissolution of the Fn clot and retraction of the keratinocyte wound layer.

## Discussion

Herein, we provide the first real-time observation of how the skin-trophic invasive GAS strain, AP53R^+^S^−^, produces a localized proteolytic environment on the bacterial surface *via* conversion of hPg to hPm. This results in significant alteration of epithelial wounds and Fn during an infection. These RTLI studies reveal for the first time that adherence and growth of GAS at leading edges of monolayer wounds are critical for local hPg activation by GAS and subsequent rapid retraction of the keratinocyte layer. When the activation of hPg by GAS was inhibited by the individual deletion of the GAS virulence factors PAM and SK, rapid wound retraction did not occur. Therefore, the retraction of wounds by GAS during an infection is dependent on the localized recruitment and conversion of hPg to hPm by the GAS virulence factors, PAM and SK. Due to the lack of difference in keratinocyte cell death with or without the activation of hPg, general cytotoxicity to keratinocytes did not play a role in the retraction of the wound when GAS and hPg were present. This demonstrates that dynamic retraction of the open wound by GAS is hPg activation-dependent. Additionally, this study reveals that our invasive strain of GAS can target and manipulate integrin β1 of keratinocytes to promote cellular detachment and likely cause wound retraction. Our studies demonstrate that following hPg activation by GAS, integrin β1 is no longer present in high abundance on the basal and apical side of the cells. GAS causes integrin β1 relocalization to the center of the cells, compromising the ECM, and leading to retraction of the epithelial layer at the leading edge of the wound.

Injury of the protective skin layer provides access for GAS to establish an infection below the layer of the dermis, and subsequently modulate the innate immune responses to gain access to deeper tissues for subsequent dissemination. The coagulation system serves a critical role in skin-wound based infections of the host and provides an immediate Fn clot matrix that contains the wound and immobilizes pathogens for detection by immune systems (3, 8, 9, 42). We sought to monitor how GAS targets a Fn clot for dissolution in RTLI of GAS-infected fluorescent-labeled Fn clots. To do so, studies were performed with fluorescent-labeled Fn clots to examine different components of the coagulation pathways. Previously, fluorescent-labeled clots were used to demonstrate crosstalk between the coagulation and fibrinolysis systems *via* activated platelets controlling both clot formation and lysis (43). In another study, fluorescent-labeled clots revealed that thrombin-activatable fibrinolysis inhibitor (TAFI) targets activated platelets to mitigate fibrinolysis (44). A scarcity of studies involving RTLI of bacteria and labeled Fn have been published. In one instance, Fn clots showed that Fn attenuates bacterial migration and dissemination at a wound and injury site (45). For our purposes, RTLI of GAS-dependent clot dissolution revealed a temporal dissolution of Fn that occurred rapidly at ~2 h p.i. As evidenced by the real-time imaging, and confirmed by clot fluorescence measurements, there was a sudden and precipitous dissolution of the clot. This indicated a highly coordinated event by GAS to attack and dissolve the Fn clot during infection.

Our combination clot and wound model showed that GAS initially dissolves the clot prior to retraction of the keratinocyte wound. This is a critical finding in understanding how skin-trophic strains of GAS initiates an infection at the wound site and progresses to dissemination. In some strains, GAS is known to form complexes with fibrinogen (Fg) *via* its particular M-Protein (46) and, in the case of the AP53R^+^S^−^ strain of GAS, Fg does not directly bind to PAM, but can bind to the AP53R^+^S^−^/PAM/Pg complex, since hPg binds to PAM *via* hPg kringle (K)2 and Fg binds to hPg *via* K1, K4, and K5 (11, 13, 47–49). Once GAS is trapped by Fg, different leukocytes are recruited to the wound in order to eliminate the bacteria. The GAS/(hPg)Fg complex is able to bind to neutrophils and induces neutrophils to release heparin binding protein (46). Heparin binding protein can cause vascular leakage and permeability, which allows for easier conditions for GAS to spread (50). Once conditions are more favorable for invasion, GAS can rapidly dissolve the clot and allow for dissemination to other parts of the host. While we did not see retraction of the wound in our combination model of wound and clot, the high load of bacteria observed post-fibrinolysis would indicate that shortly thereafter it targets the epithelial wound interface at the keratinocyte, as seen in our wound models. In the future, we endeavor to increase the complexity of our model by building a 3-D based tissue model system that incorporates fibroblast, keratinocyte and endothelial cells in order to build a true “skin layer” system to observe host-pathogen interactions.

Our RTLI studies reveal critical temporal insights into how a highly invasive skin-trophic GAS strain targets epithelial wounds and fibrin clots for dissemination from an initial breach in the host skin barrier. Our combined *in situ-*based cellular model of fibrin clot-in epithelial wound to simulate the progress of bacterial infections at the skin barrier may provide insights into how general skin pathogens can dynamically alters host environments during dissemination and invasion. Our RTLI model may be utilized in other areas of research involving general cellular injury and the hemostatic process.

## Data Availability Statement

The original contributions presented in this study are included in the article and Supplemental Materials section. Further inquiries can be directed to the corresponding author.

## Author Contributions

HV, DLH, AF, VP, FC, and SL: designed the overall project and experimental aims. HV, DEH, ZL, MB, GN, CP, and TM: performed experimental work and analyzed the results. HV and SL: wrote the paper, with section contributions from DEH, VP, and FC. All authors contributed to the proofreading and editing of the paper.

## Conflict of Interest

The authors declare that the research was conducted in the absence of any commercial or financial relationships that could be construed as a potential conflict of interest.
