# Supplementary material for: Group A Streptococcus-Induced Activation of Human Plasminogen Is Required for Keratinocyte Wound Retraction and Rapid Clot Dissolution
Source: Front Cardiovasc Med. 2021 Jun 10;8:667554. doi: 10.3389/fcvm.2021.667554 (PMC8230121; doi:10.3389/fcvm.2021.667554)
Supplement: Supplementary file 1 [file Data_Sheet_1.PDF]

Supplementary Material

Supplemental Movies 1-10

Supplemental Movie 1: Activation of hPg by AP53R^+S^- causes wound retraction of keratinocytes

Supplemental Movie 2: Plasmin causes a rapid wound retraction of keratinocytes

Supplemental Movie 3: AP53R^+S^- does not induce retraction of a keratinocyte wound in the absence of hPg

Supplemental Movie 4: hPg does not cause keratinocyte wound retraction in the absence of GAS

Supplemental Movie 5: Activation of hPg by AP53R^+S^- causes Fn clot lysis

Supplemental Movie 6: AP53R^+S^- does not lyse Fn clot in the absence of hPg

Supplemental Movie 7: Combination clot-in-wound model infected with GAS AP53R^+S^- and hPg shows hierarchical rapid clot dissolution

Supplemental Movie 8: AP53R^+S^-/ΔSK does not retract a keratinocyte wound

Supplemental Movie 9: AP53R^+S^-/ΔPAM does not retract a keratinocyte wound

Supplemental Movie 10: GAS AP53R^+S^-/ΔSK does not dissolve fibrin clots

Supplementary Figures

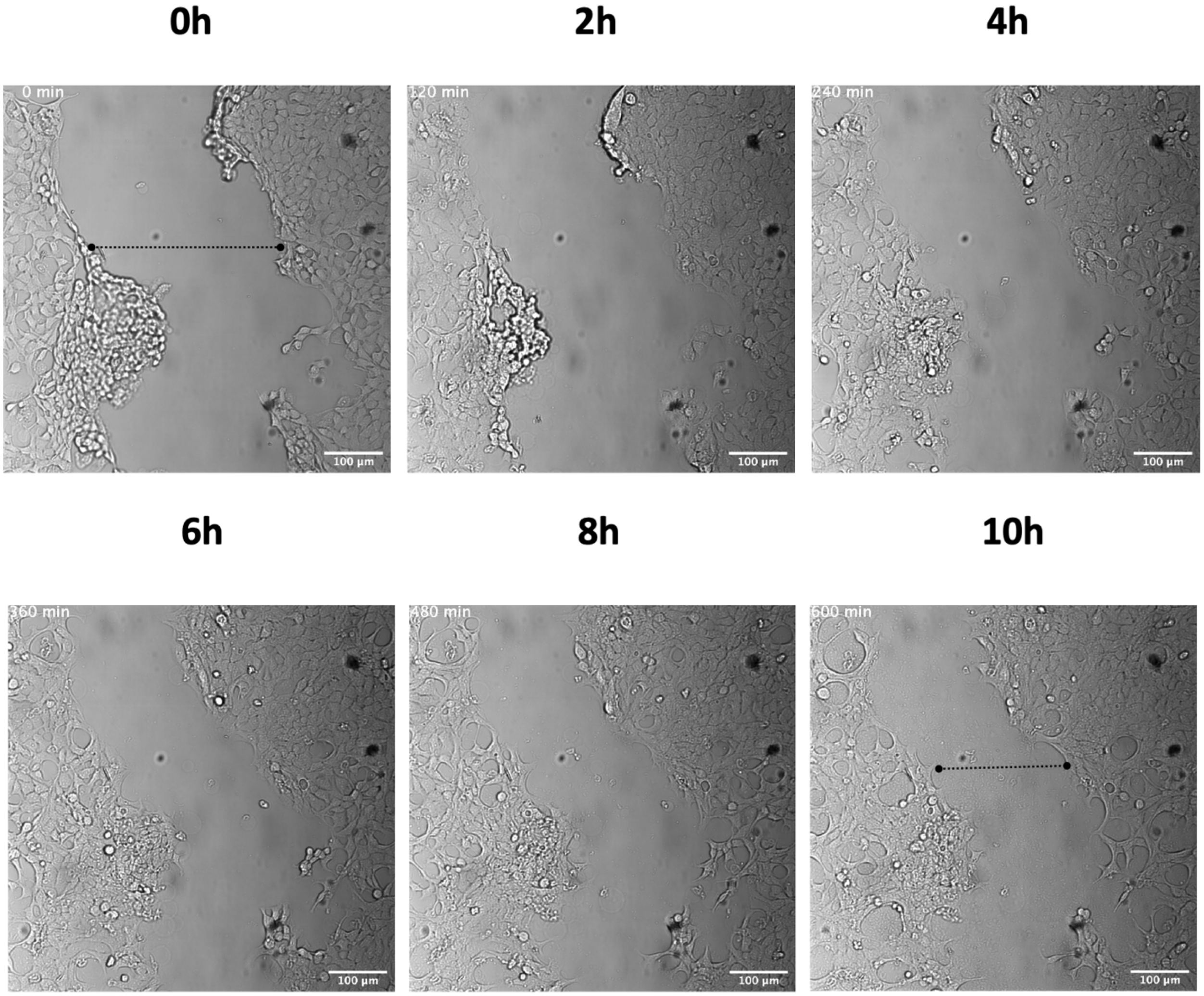

Supplementary Figure 1: AP53R^{+}S^{-}/ΔSK did not retract a keratinocyte wound. Scratch wounds were made as in Figure 1. The cells were then incubated with AP53R^{+}S^{-}/ΔSK at an MOI 5, along with the addition of 7 µg/ml hPg prior to live imaging. Images were obtained every 10 min for 10 hr. Time-lapsed images of experiment are shown here (See Supplemental movie 8). The dashed lines show the approximate width of the wound at the start and the end of the experiment.

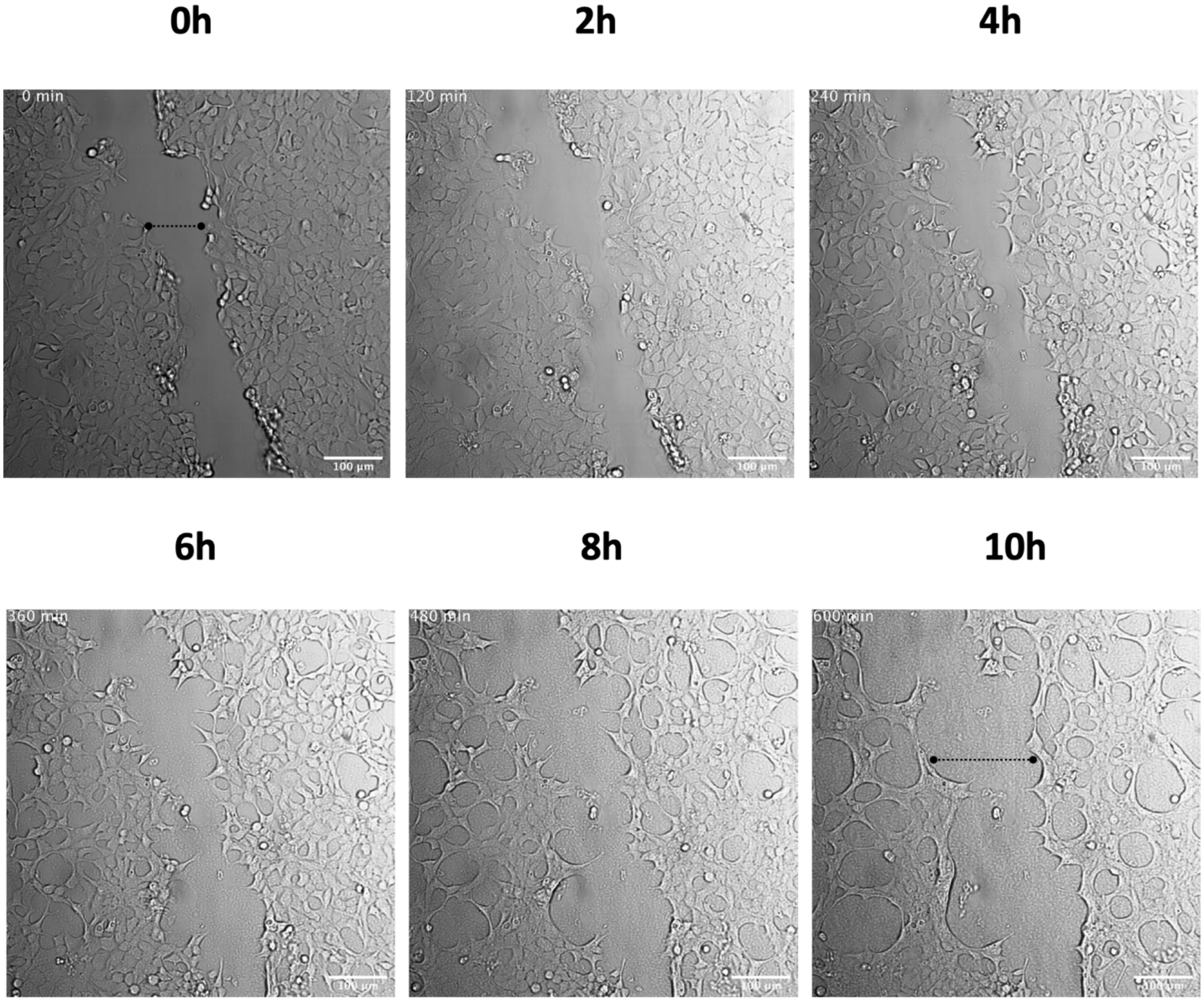

Supplementary Figure 2: AP53R^{+}S^{-}/ΔPAM did not retract a keratinocyte wound. Scratch wounds were made as in Figure 1. The cells were then incubated with AP53R^{+}S^{-}/ΔPAM at MOI 5 along with the addition of 7 μg/ml hPg prior to live imaging. Images were obtained every 10 min for 10 hr. Time-lapsed images of experiment are shown here (See Supplemental movie 9). The dashed lines show the approximate width of the wound at the start and end of the experiment.

0h Wound

A

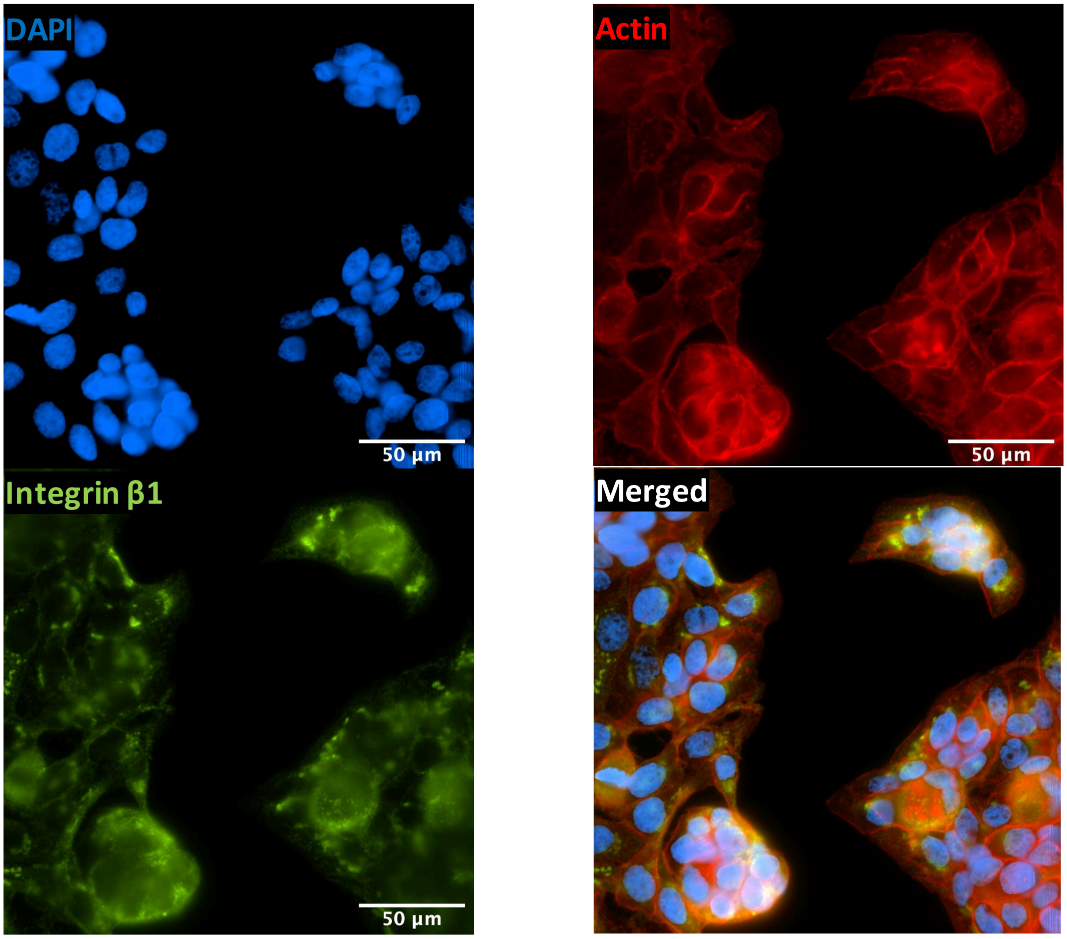

0h Z-Stack Cross-Section

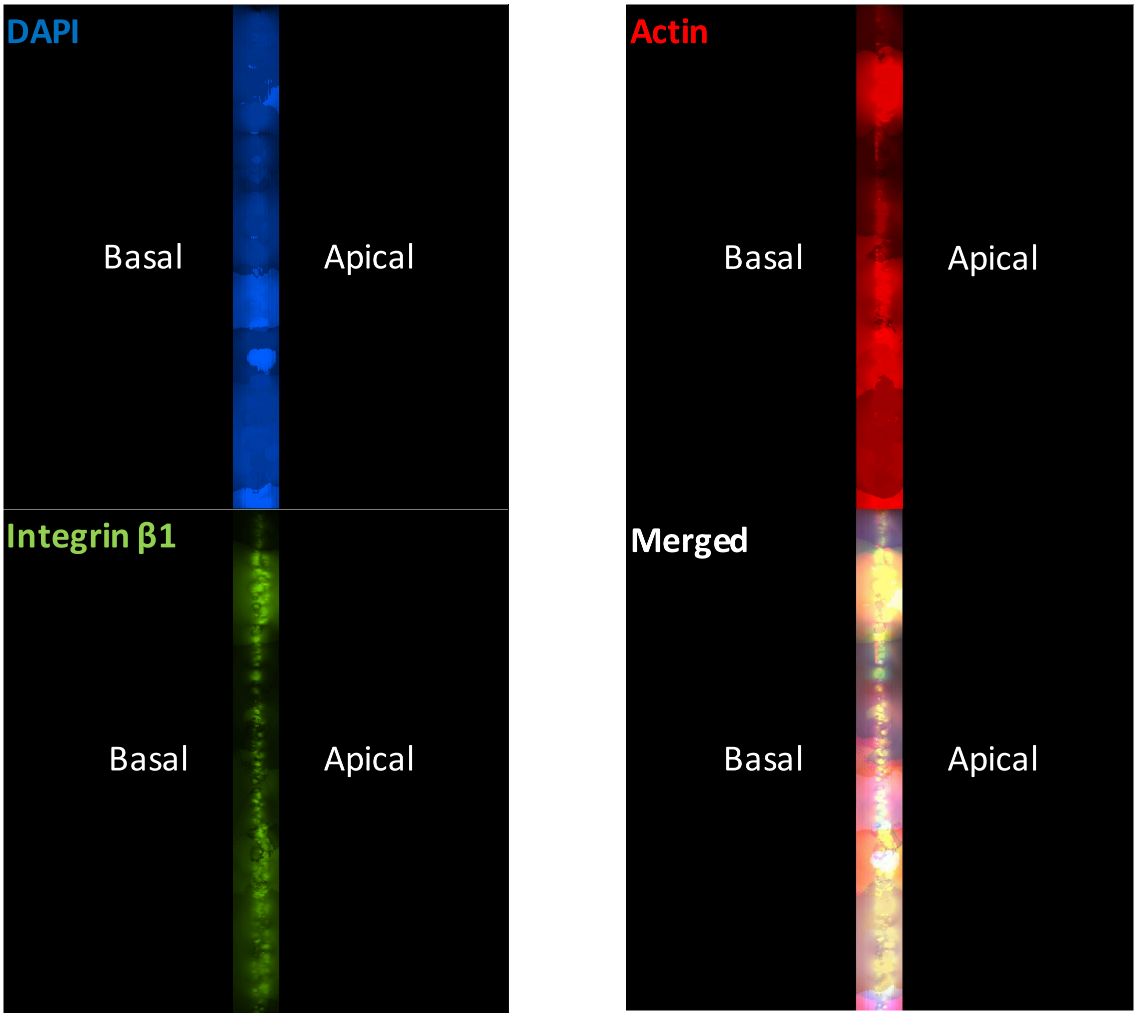

B

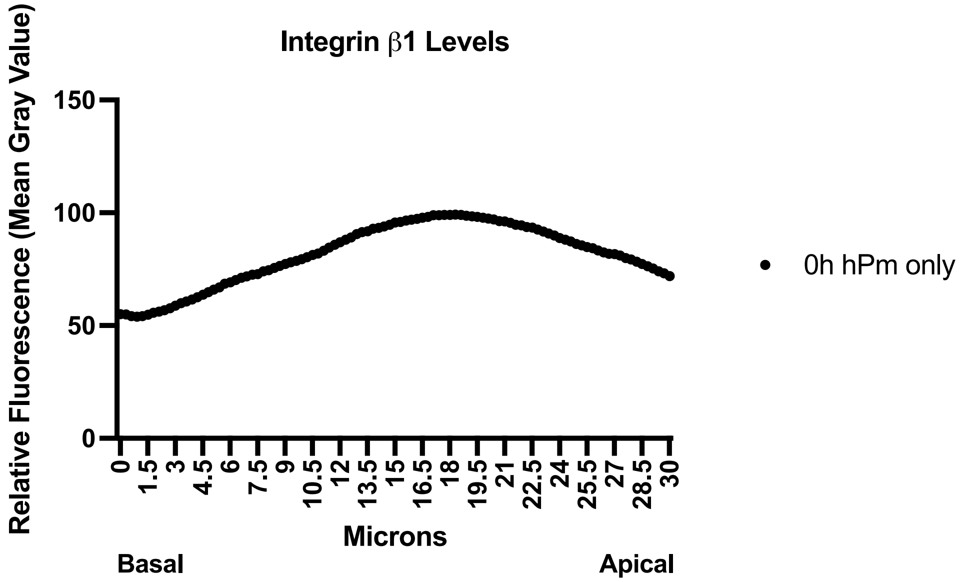

Supplementary Figure 3: hPm promotes relocalization of integrin ß1 in a keratinocyte wound. Scratch wounds were produced as in Figure 1. (A) Cells were incubated with hPm (7 µg/ml) prior to fluorescence imaging. (B)Fluorescence readings for an antibody to integrin ß1were analyzed for each image via ImageJ/Fiji after hPm addition. The red circles indicate aggregate fluorescence readings at 0 hr after hPm addition. The images were obtained every 0.3 µm for 30 µm beginning at the basal level.

0h Wound

A

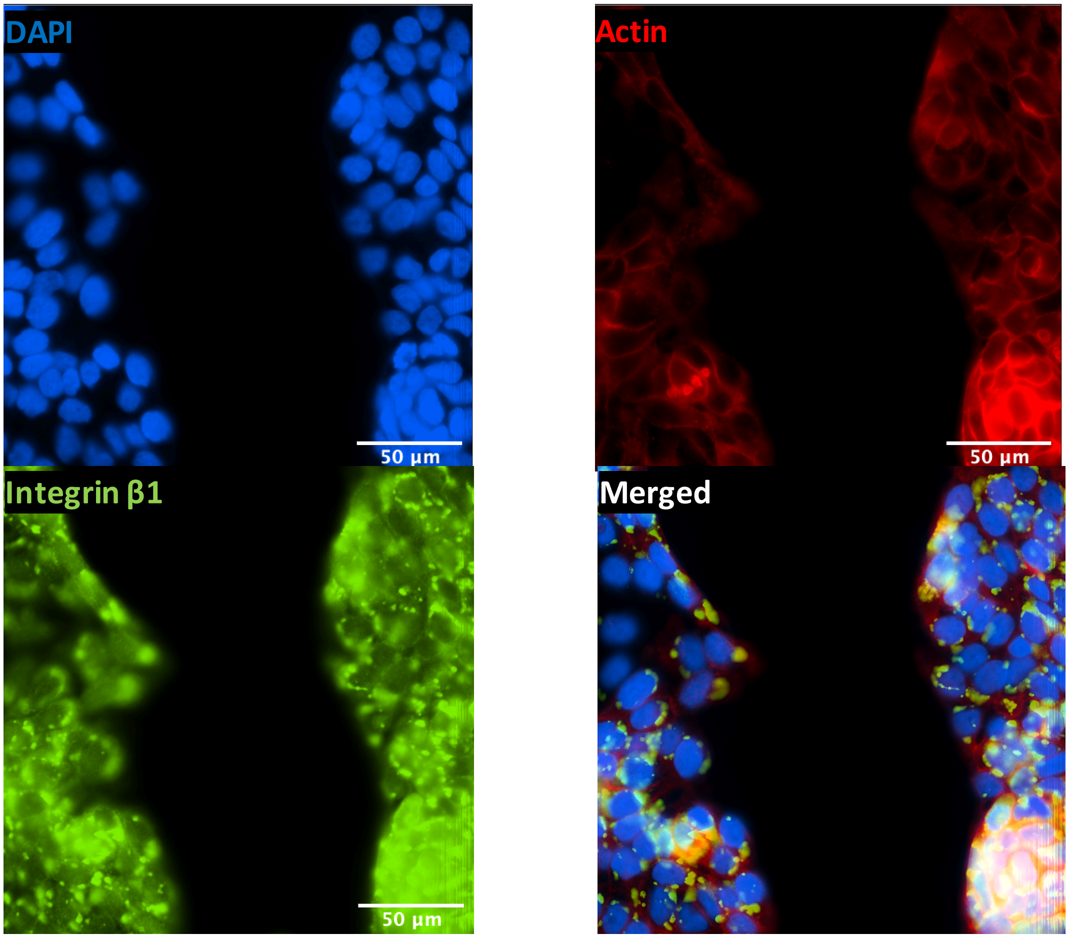

0h Z-Stack Cross-Section

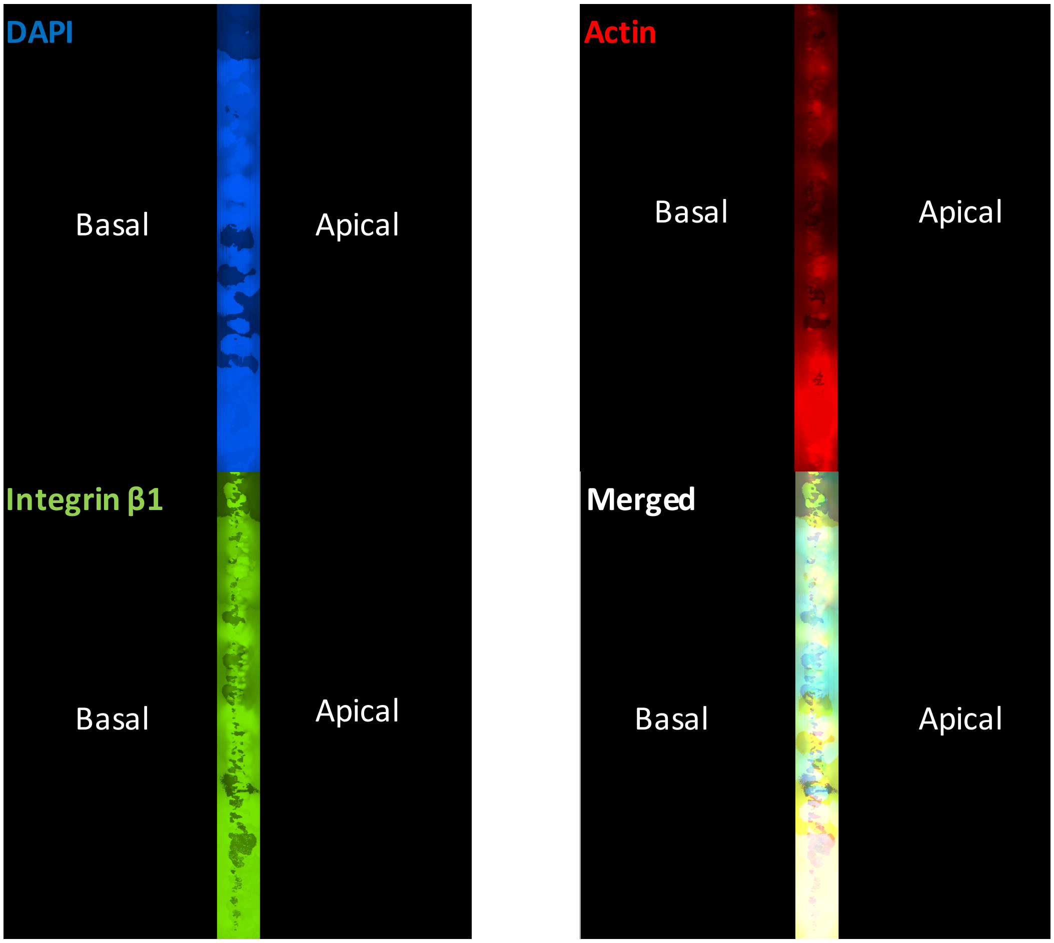

8h Wound

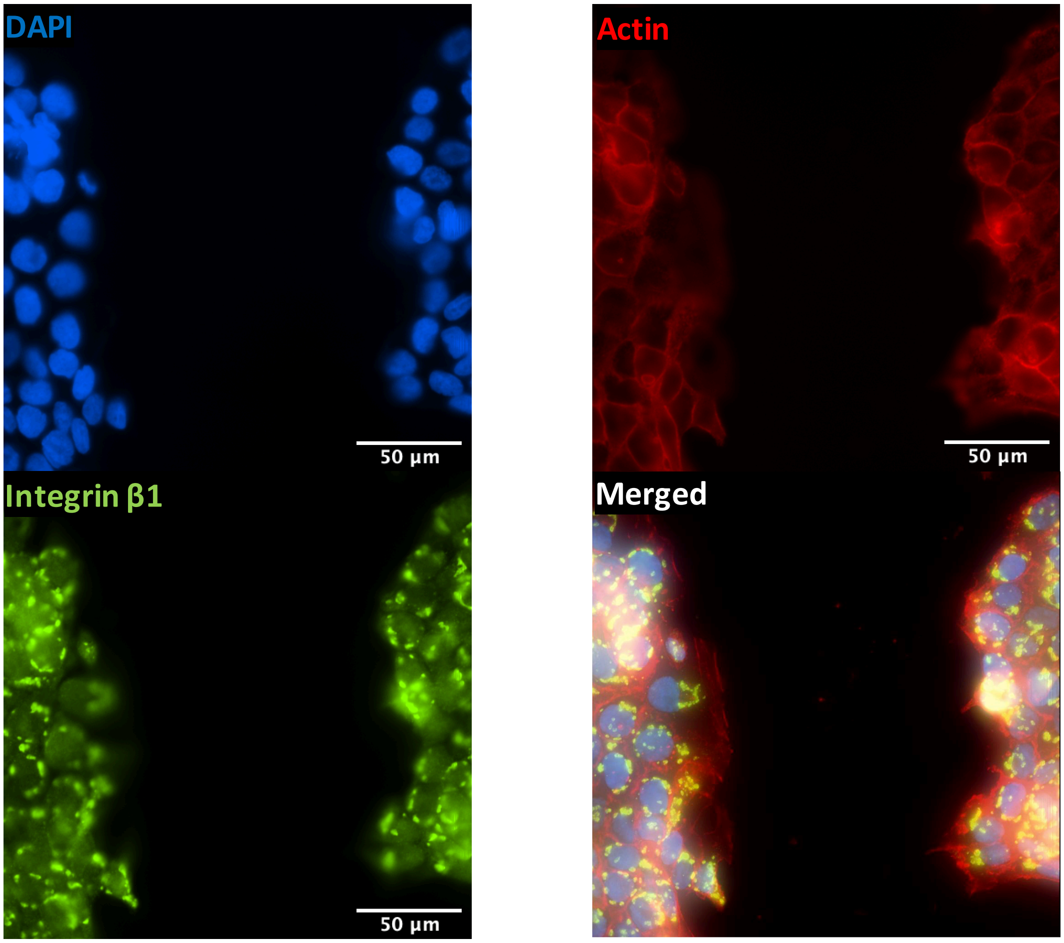

8h Z-Stack Cross-Section

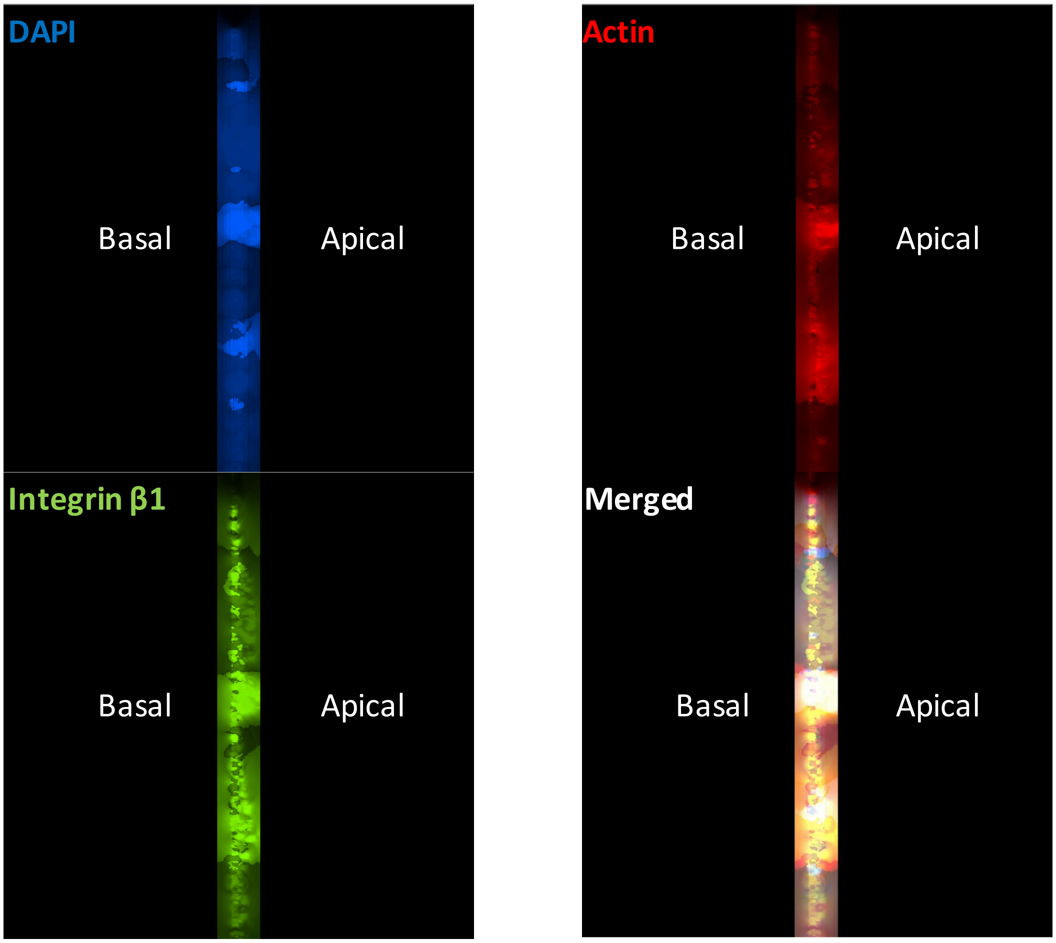

B

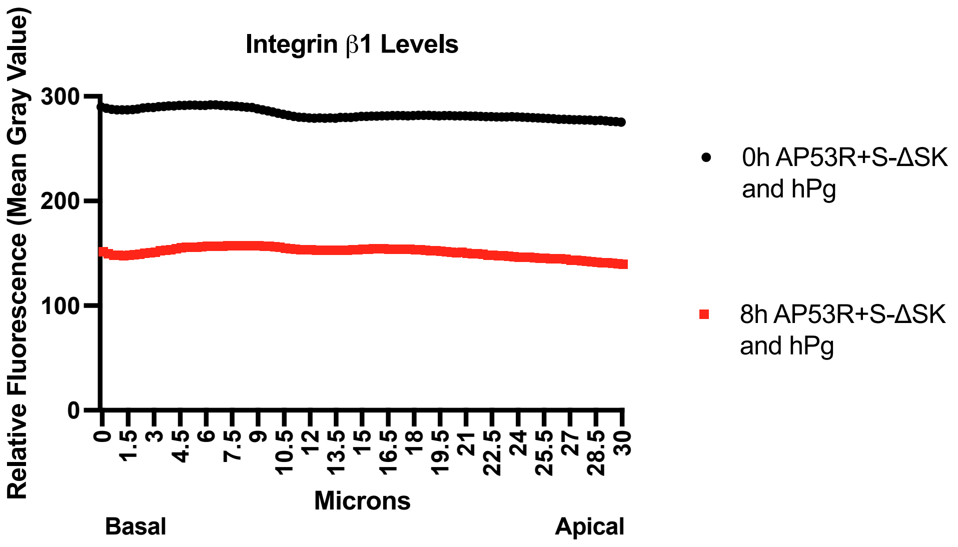

Supplementary Figure 4: AP53R$^+$S$^-$/ΔSK did not affect integrin ß1 localization in a keratinocyte wound. Scratch wounds were produced as in Figure 1. (A) The cells were incubated with AP53R$^+$S$^-$/ΔSK along 7 µg/ml hPg prior to imaging at 0 hr and 8 hr. (B) Fluorescence readings for anti-Integrin ß1were analyzed for each image via ImageJ/Fiji after infection with GAS and hPg. The black circles indicate aggregate fluorescence readings at the initial infection. The red circles indicate aggregate fluorescence readings at 8 hr after infection. Images were obtained every 0.3 µm for 30 µm beginning at the basal level.

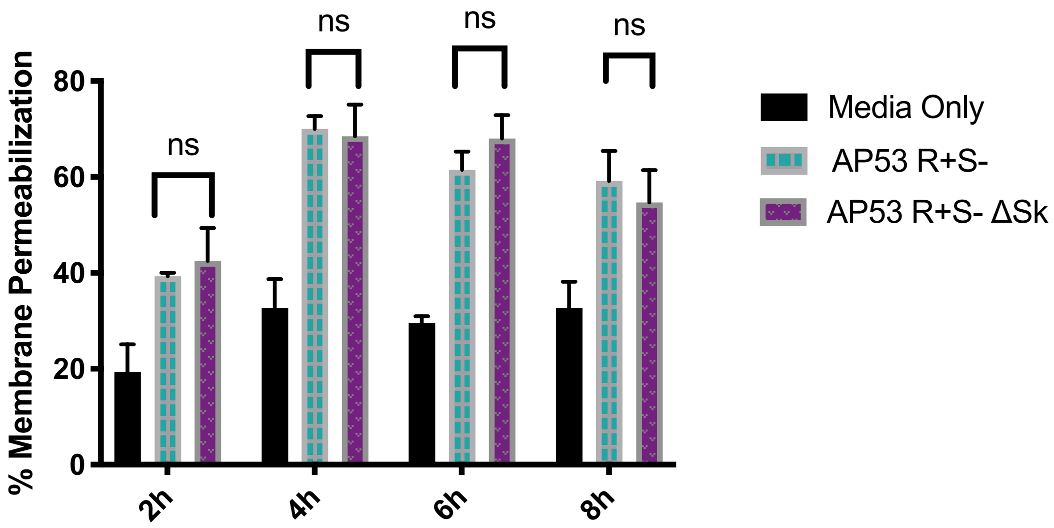

Supplementary Figure 5: Infections with hPg and AP53 R+S$^-$ as well as with AP53 R$^+$S$^-$/ΔSK did not reveal a difference in cell viability. Over the course of 8 hr, in the presence of 100 µg/mL hPg, there was no difference in cell viability when HaCaT cells were infected with AP53 R+S$^-$ and its isogenic mutant AP53 R$^+$S$^-$/ΔSK.

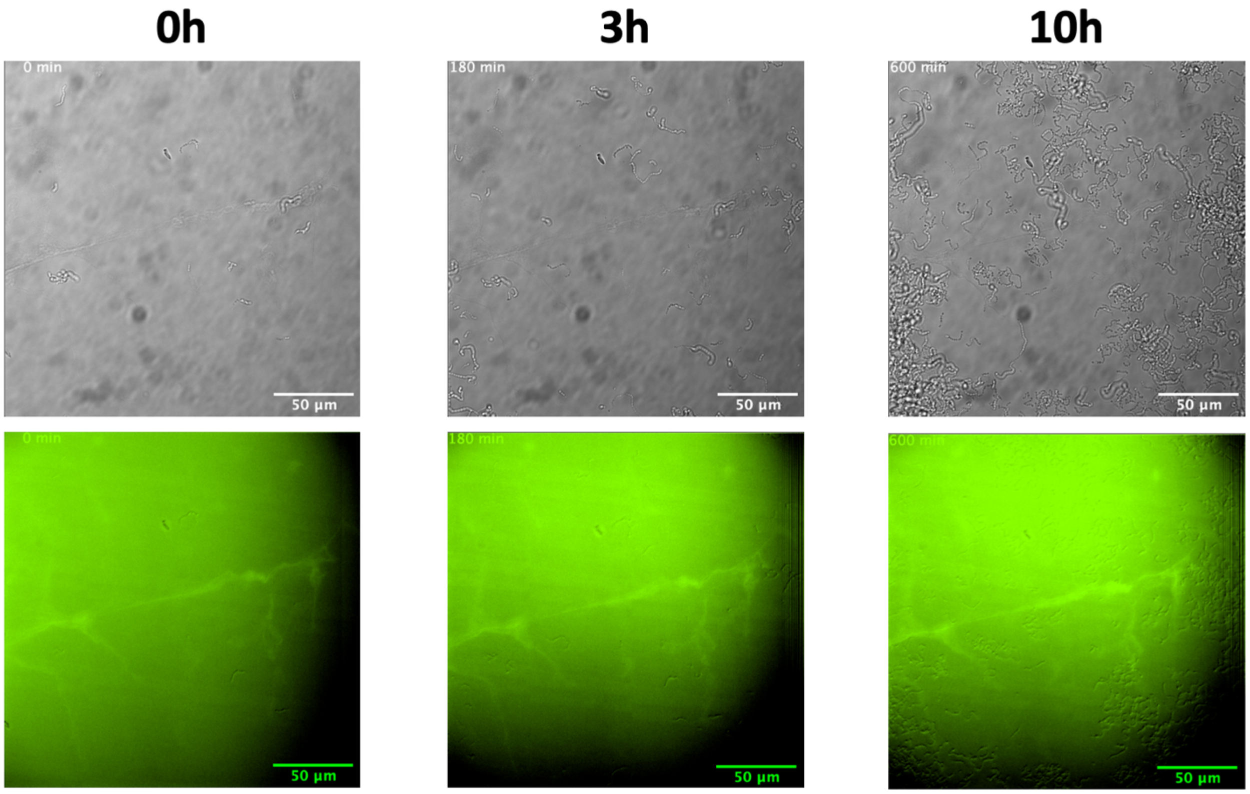

Supplementary Figure 6: AP53R$^+$S$^-$/ΔSK did not dissolve the Fn clot. Prior to live imaging, the fibrin clot was incubated with AP53 R$^+$S$^-$/ΔSK at an MOI of 5 along with 7 µg/mL of hPg. Images were obtained every 10 min for 10 hr for the duration of the imaging experiment. Time-lapsed images of experiment are shown here (See Supplemental movie 10).
